# Marine Benthic Diatoms Contain Compounds Able to Induce Leukemia Cell Death and Modulate Blood Platelet Activity

**DOI:** 10.3390/md7040605

**Published:** 2009-11-18

**Authors:** Siv Kristin Prestegard, Linn Oftedal, Rosie Theresa Coyne, Gyrid Nygaard, Kaja Helvik Skjærven, Gjert Knutsen, Stein Ove Døskeland, Lars Herfindal

**Affiliations:** 1 Department of Biology, University of Bergen, Jahnebakken 5, N-5020 Bergen, Norway; E-Mail: gjert.knutsen@bio.uib.no (G.K.); 2 Department of Biomedicine, University of Bergen, Jonas Lies vei 9, N-5009 Bergen, Norway; E-Mails: linn.oftedal@biomed.uib.no (L.O.); rosie.coyne@biomed.uib.no (R.C.); gyrid.nygard@biomed.uib.no (G.N.); ksk@nifes.no (K.S.); stein.doskeland@biomed.uib.no (S.D.); lars.herfindal@biomed.uib.no (L.H.); 3 Proteomic Unit at the University of Bergen, Jonas Lies vei 9, N-5009 Bergen, Norway

**Keywords:** adenosine, *Amphora* sp., apoptosis, bioactivity, blood platelets, diatoms, drug discovery, Phaeodactylum tricornutum, *Melosira* sp., natural products, Nitzshia pusilla

## Abstract

In spite of the high abundance and species diversity of diatoms, only a few bioactive compounds from them have been described. The present study reveals a high number of mammalian cell death inducing substances in biofilm-associated diatoms sampled from the intertidal zone. Extracts from the genera *Melosira*, *Amphora*, *Phaeodactylum* and *Nitzschia* were all found to induce leukemia cell death, with either classical apoptotic or autophagic features. Several extracts also contained inhibitors of thrombin-induced blood platelet activation. Some of this activity was caused by a high content of adenosine in the diatoms, ranging from 0.07 to 0.31 μg/mg dry weight. However, most of the bioactivity was adenosine deaminase-resistant. An adenosine deaminase-resistant active fraction from one of the extracts was partially purified and shown to induce apoptosis with a distinct phenotype. The results show that benthic diatoms typically found in the intertidal zone may represent a richer source of interesting bioactive compounds than hitherto recognized.

## 1. Introduction

The diatoms represent a large and extraordinary ecologically flexible group of unicellular eukaryotic photosynthetic microalgae. The species diversity of diatoms is large, and estimates range from 1 × 10^4^ [1] to 2 × 10^5^ species [2]. They have genes from the plant, bacteria and animal kingdom, and this has resulted in a unique metabolism [3].

The best-described diatom bioactive compound is the neuroexcitatory amino acid derivative domoic acid. Human consumption of domoic acid-contaminated mussels during a bloom of *Pseudo-nitzschia multiseries* has caused mass poisoning [4]. There is also emerging evidence that the oxidative transformation of fatty acids to reactive unsaturated aldehydes and hydrocarbons in certain planktonic diatoms can impact the survival and reproduction of diatom grazers like copepodes and kill human colon adenocarcinoma cells [5,6]. Anther bioactive compounds reported from diatoms is naviculan, isolated from *Navicula directa* [7], a sulfated polysaccharide with antiviral activities against herpes simplex viruses 1 and 2 and influenza A virus.

In view of the metabolic capabilities and diversity of diatoms it is enigmatic why so few bioactive compounds have been described. We have previously reported that benthic cyanobacteria have a much higher frequency and variation of cytotoxic compounds than planktonic cyanobacteria [8,9]. Our objective was thus to find if benthic diatom communities represent a promising source for bioactive compounds. We therefore tested extracts from unicellular benthic diatoms from the intertidal zone, associated onto surfaces like rocks (epilithic), sand (episamnic) or plants (epiphytic) for ability to kill acute myelogenic leukemia cells and to modulate blood platelet activation induced by thrombin. Agents able to modulate these functions without side effects are obvious drug candidates for cancer therapy and control of cardiovascular diseases, respectively. We also included primary rat hepatocytes in our screening, since we have previously shown that these can be used to detect novel toxins [9], however not useful as anti-cancer drug candidates. We report that a surprisingly high proportion of the benthic diatom isolates had cytotoxic and blood platelet inhibitory activity, as well as unusually high content of adenosine. Benthic diatoms from the intertidal zone appear therefore to be an underexplored source of bioactive compounds.

## 2. Results

### 2.1. Marine benthic diatoms are a rich source of leukemia cell death inducing activity

Biomass from ten diatom isolates was extracted sequentially, and the extracts were tested for ability to induce hepatocyte and leukemia cell death. We found strong induction of leukemia cell death by seven of the aqueous extracts. Only three aqueous extracts induced hepatocyte cell death (Table 1). While several of the methanol extracts showed moderate leukemia cell death induction, none of the organic extracts showed any death induction (Table 1).

A subclone of the IPC leukemia cells expressing the prosurvival oncogenic protein Bcl-2 is resistant towards several apoptotic stimuli [10,11], including current anti-leukemic chemotherapy drugs like the anthracycline. Although the IPC-Bcl-2 cells were completely resistant to some extracts (from isolates ND 50, 51, 53, 71 and 79) they were only partly resistant to extracts from isolates ND52, 55, 56, 58, 73 (Table 1). This is of interest since an important challenge in cancer therapy is to overcome treatment resistance due to cancer cell overexpression of survival proteins like Bcl-2. A well-known group of microbial toxins are protein phosphatase targeting substances like microcystin, nodularin, calyculin A and okadaic acid [12]. These are also able to overcome the cell death protection by Bcl-2 [13], but we found no such activity in any of the extracts (not shown) using a highly sensitive assay [14].

**Figure 1.**
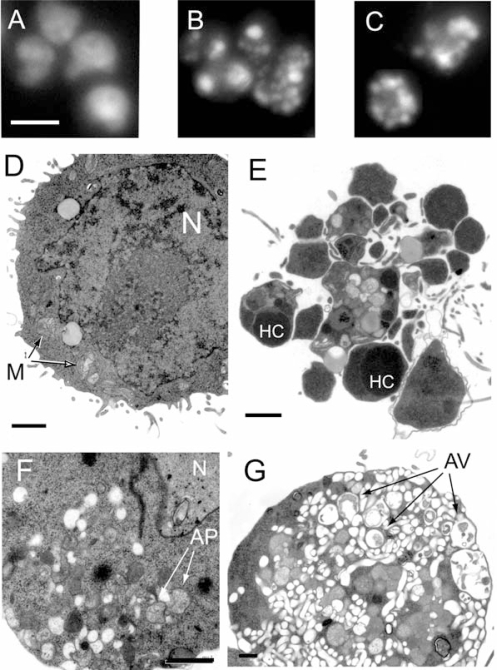
Morphology of cells after treatment with diatom extracts. (A–C) Fluoresence microscopy of IPC-81 cells incubated with aqueous diatom extract (4 mg/mL) for 24 hours, fixed with formaldehyde, and the DNA stained with Hoechst 33342. (A) Control cell, (B) ND52, (C) ND58. (D–G) Transmission electron microscopy of cells treated with solvent (D) or aqueous extract of ND58 for 8 hours (E–G). Panel (E) shows a cell with typical apoptotic morphology, panel (F) a cell with early signs of autophagy, and panel (G) a cell with extensive autophagy. N: Nucleus, M: Mitochondria, HC: Hypercondensed chromatin, AP: Auto-phagosomes, AV: Autophagic vesicles. Scale bar is 10 μm in (A–C) and 1 μm in (D–G).

**Figure 2.**
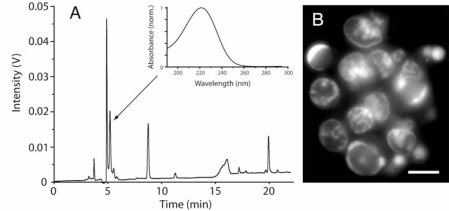
Purification of an apoptogenic activity in diatom strain ND73 (*Nitzschia cf. pusilla*). (A) Anion exchange extract was separated by reversed phase HPLC (Section 4.7) and fractions tested for toxicity. The 5–6 min fraction contained a peak (5.5 min, inset shows UV-spectrum) that induced a morphology characterized by chromatin margination (B) after 24 h incubation with same concentration as in Figure 1B and D. Scale bar: 5 μm.

During the screening we discovered that the extracts produced distinct forms of cell death. The cell death morphology was therefore studied in more detail (Figures 1A–C, Figure 2B). When tested at a concentration inducing about 50% overall cell death, the aqueous extract from ND73 induced chromatin margination without alteration of the nuclear shape or fragmentation of the nucleus of IPC-81 cells (Figure 2B). In contrast, the other aqueous extracts produced massive nuclear fragmentation (Figures 1B and C). At higher concentrations the extracts caused complete nuclear disintegration (not shown). An in depth morphological analysis by transmission electron microscopy was undertaken of IPC-81wt cells treated with the ND58 aqueous extract (Figures 1 D–G). IPC-81wt cells treated with ND58 aqueous extract for 8 h showed two distinct morphologies. A typical apoptotic cell (Figure 1E) had disintegrated into apoptotic bodies, which could contain nuclear fragments with hypercondensed chromatin, mitochondria, and other organelles. In some cells, we noted the formation of vesicles resembling autophagosomes (Figure 1F), probably representing an early stage towards excessive autophagy where the cell interior was dominated by autophagic vesicles containing partially digested organelles (Figure 1G). The nucleus appeared to be disintegrated, and the nuclear envelope and chromatin could not be identified.

**Figure 3.**
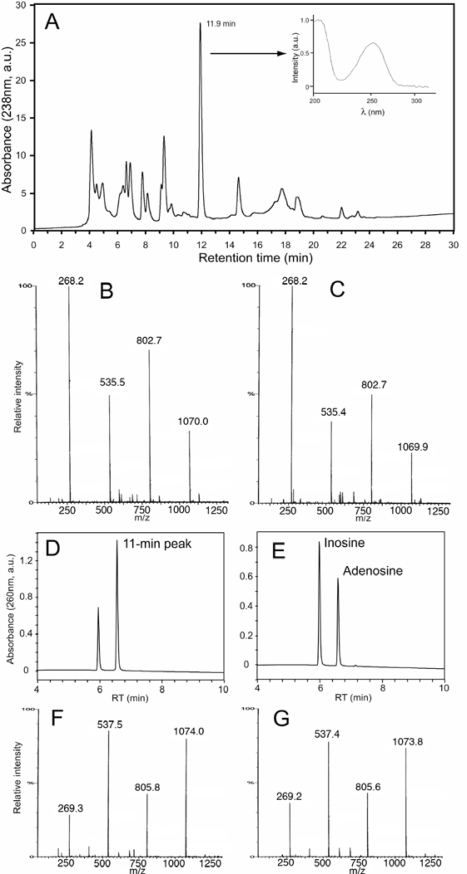
Identification of adenosine as one of the anti-leukemic activities in ND58. (A) Aqueous extracts was passed through an anion exchange cartridge and subjected to reversed phase HPLC. The anti-leukemic activity eluted in a peak at 11.9 min (UV-spectrum is shown in inset). (B–C) Comparative MS analysis of the compound of the 11.9 min peak (B) with adenosine (C). (D–E) Reversed phase HPLC of the 11.9 min peak (D) and adenosine (E) after treatment with adenosine deaminase. (F–G) MS analyses of the deaminase degradation product of the 11.9 min peak (F) and inosine (G) after treatment with adenosine deaminase.

We next wanted to find out if different toxins caused the cell death morphologies induced by ND58 (Figure 1C) and ND73 (Figure 2B). We found that reversed phase chromatography of an anion exchange extract from ND73 gave a toxic fraction at around 5 minutes (Figure 2), which reproduced the distinct nuclear morphology seen in cells treated with the crude extract (Figure 2B). In contrast, the toxicity of ND58 eluted later under identical chromatographic conditions (Figure 3A and data not shown). We have not been able to purify enough material from ND73 to continue the analyses of the toxic fraction, but noted that the UV-spectrum of the active fraction of ND73 (inset in Figure 2A) was different from that of ND58 (inset in Figure 3A). Moreover, the anion exchange extract of ND73 produced a peak at 8 min (Figure 2A) with similar UV-spectrum (not shown) as that of the active peak of ND58 (inset in Figure 3A). We conclude that the diatoms contained water-soluble compounds able to induce various types of cell death in leukaemia cells, including classic apoptotic and autophagic death, both via pathways blocked by Bcl-2 and pathways overriding Bcl-2.

### 2.2. Diatoms may contain unusually high level of adenosine and other adenosine deaminase-sensitive compounds

Some of the anti-leukemic activity of the ND58 extract was irreversibly bound to the QMA-resin during solid phase separation, and less than half of the initial activity was recovered in the flow-through and wash fraction. This apoptosis-inducing fraction was loaded onto semipreparative reversed phase HPLC, and nearly all the cytotoxic activity was found to be associated with one peak (Figure 3A). When analysed by Q-tof mass spectrometry (Figure 3B and C) the peak was found to contain multimers of m/z 268 (Figure 3B), which when fragmented all produced a daughter-ion with m/z of 136 and a corresponding neutral loss of 132 Da (not shown), like adenosine (Figure 3C). When exposed to adenosine deaminase the activity was lost and a compound appeared with the retention time (Figure 3D and E) and mass of inosine (Figure 3F and G). We conclude that part of the antileukemic activity in the ND58 extract could be ascribed to adenosine. This was surprising in view of the high dilution of the extracts used for screening, and led us to determine the adenosine content in fresh samples of the various diatom strains. We found that while isolate ND58 contained 0.17 μg adenosine per mg dry weight, four isolates contained even more (like ND73: 0.31 μg/mg dry weight), while two isolates (ND53 and ND 71) had very little adenosine (Table 2).

In order to better assess the importance of adenosine and related metabolites for diatom toxicity, adenosine deaminase was added to the aqueous extracts and they were then rescreened for antileukemic activity (data not shown). The adenosine deaminase was active in the extract, and abolished the adenosine peak and produced an inosine peak in HPLC-chromatograms of the extracts (not shown). Deaminase treatment only decreased significantly the cytotoxic activity in extracts ND53 and ND79. This was puzzling since ND53 had very little adenosine (Table 2), and suggested that these diatoms had other adenosine deaminase-sensitive toxins. A number of such toxins have been reported in microorganisms or have been produced synthetically, and we have shown previously that some of them can induce cell death [15,16].

The LC_50_ value of adenosine is 7 μM in IPC-81wt cells. The highest adenosine level found in our diatom strains was 0.31 μg/mg biomass DW, corresponding to 4.5 μM at the highest concentrations used in the cell assay. This is enough to produce only 20% cell death (not shown). We conclude that although diatoms can contain high levels of adenosine and other toxic adenosine deaminase sensitive metabolites, most of them owe their antileukemic activity to other types of molecules.

### 2.3. Screen for platelet activation modulating activity in diatom extracts

We next tested if the aqueous diatom extracts could modulate blood platelet activation by the thrombin agonist TRAP (thrombin receptor agonist peptide). Extract from strain ND53 activated the blood platelets. The activation was insensitive to adenosine deaminase treatment (Table 2), suggesting it was due to another compound than the one responsible for IPC cell death induction, which was deaminase sensitive (see the preceding paragraph).

The other extracts produced strong to moderate inhibition of platelet activation (Table 2). Adenosine deaminase treatment abolished the inhibitory activity only for extracts ND73 and ND79 (Table 2). Since adenosine deaminase failed to abolish the antileukemic activity of extract ND73 the platelet inhibitory and antileukemic activity in this extract presumably resided in different molecules.

### 2.4. Taxonomy of the diatom isolates

The fact that diatoms previously have been only sparsely associated with production of bioactive compounds made us determine the identity of our richly bioactivity producing isolates. We employed a genetic approach by sequencing parts of the large subunit (LSU, 28S rDNA) from the isolates, and sequences were aligned together with sequences from other pennate diatom species. Since relatively few sequences from pennate diatoms are available at GenBank, we also studied the diatom morphology in detail (a thorough description of the methodology and morphology and taxonomy of the strains is given in the supplementary data). The identification of the isolates is presented in Table 2.

## 3. Discussion

Little is known about bioactive substances in diatoms, as compared to other abundant aquatic microorganisms like cyanobacteria and dinoflagellates [17]. Diatoms are responsible for massive blooms, but with the notable exception of domoic acid, these blooms are generally considered nontoxic. It was therefore surprising that all our diatom isolates from the intertidal zone from various parts of the coast of Norway showed significant cell death inducing activity towards the IPC-81 myelogenic leukemia cells (Table 1). Many isolates contained also antithrombogenic substances (Table 2). Although the isolated diatoms had a high content of adenosine the bioactivity was due mainly to compounds that were resistant to adenosine deaminase. However, the active fraction detected in ND73 (5-min peak, Figure 2) was not detected in any other diatom isolate (Figure 3A and data not shown), suggesting the presence of at least two different adenosine deaminase resistant bioactivities. One that failed to elute from the anion-exchange cartridge (e.g., ND58), and one polar compound that eluted early during reversed phase HPLC (Figure 2).

The survival protein Bcl-2 is upregulated in many cancer cells, and several current antileukemic drugs like daunorubicin fail to kill Bcl-2 overexpressing cells. However, some of the diatom extracts were able to kill also leukemia cells overexpressing Bcl-2 (Table 1). We observed a mixed death phenotype on the wt cells treated with the aqueous extract from ND58 (Figure 1). The main phenotype was apoptotic (Figure 1E), but some cells showed sign of early autophagic features (Figure 1F). However, we also noted cells with morphology typical for autophagic death (Figure 1G). Moreover, the polar toxic compound found in ND73 produced a distinct death phenotype (Figure 2B), which suggests that the toxin targets different death mechanisms than those in the other isolate extracts. We believe that these are examples of interesting leads for further exploitation both regarding detailed mechanism of action and their potential as anticancer agents.

The high frequency of isolates with bioactivity in our screen could not be ascribed to sampling of one particular species since four different genera were represented (see supplementary material for a thorough discussion of the taxonomy of the isolated diatoms). Besides, the type of bioactivity in two different isolates of the same species differed with respect to sensitivity to adenosine deaminase and relative activity towards leukemia cells and thrombocytes (ND53 and 73, Tables 1 and 2). We conclude that the benthic diatoms are an abundant source for bioactive compounds. These are exposed to fluctuations in salinity, temperatures, light and nutrients, and are also subjected to gracing from predators. This may explain why they produce bioactive substances that also target important processes in mammalian cells.

The culturing of marine benthic diatoms at intermediate and high scale is not always straight forward, but we were able to establish growth conditions that allowed considerable biomass production with sustained ability to produce bioactivity over a period of several years (see supplementary material for further details). In view of the evolutionary history and the high gene diversity and metabolic pathways in diatoms, we anticipate that several unique compounds may exist in diatoms. In line with this, the present study suggests that benthic diatoms have a capacity to produce bioactive substances that should be further utilized.

## 4. Experimental Section

### 4.1. Diatom isolation and cultivation

Diatoms were isolated by dilution series of samples from microbial biofilms on rocks, sediments and marine plants in the intertidal zone along fjords on the western part of Norway (see table 1). All cultures were grown in Conway medium prepared from filtrated seawater diluted with distilled water to 80% [18]. Diatom cultures were cultured at 20 °C in 250 mL glass cylinders with conical bottom (inner diameter 3.5 cm) and humified air mixed with CO_2_ (1% CO_2_ final concentration) was filtered through bacterial filters (0.45 μm) and bubbled through the cultures. The cultures were provided with 116 μmol photons m^−2^ sec^−1^ continuous light from fluorescent tubes (Phillips). Cultures were harvested in early stationary growth phase after one week by centrifugation at 900 × g for 10 min, washed twice with water and the pellets were frozen and lyophilized. The lyophilized samples were stored at −20 °C. Water was always Milli-Q quality from Millipore systems.

### 4.2. Preparation of diatom crude extracts

Between 3 and 10 mg dry diatom biomass was measured and transferred to Eppendorf tubes and added 0.4 mL MQ water. The cells were added 0.5 mL 0.45 mm glass beads and mixed vigorously by a vortex blender at seven one-min pulses and cooled on ice between the vortex pulses. The extract was separated from the glass beads, and the glass beads were washed twice with 0.6 mL MQ water. The complete destruction of the cells was confirmed by microscopy. The combined extracts and washes were pooled and extracted on ice for 1 h before centrifugation at 1600 × g for 10 min. The supernantant was the water extract. The pellet was resuspended in 0.4 mL of ice-cold 70% aqueous methanol, left on ice for 1 h, and centrifuged and washed with 70% methanol to produce the 70% methanol extract. The pellet was extracted further with 0.4 mL ice-cold 1:1 methanol-dichloromethane for 12 h in the dark. The mixture was then centrifuged, the pellet washed twice, and the supernatants were combined to give the organic extract. The extracts were dried in a vacuumed centrifuge and resuspended in either 100 μL of MQ water (aqueous extract) or 100 μL of 25/75% dimethylsulfoxide/water (v/v). The concentrations given in the tables and figures correspond to amount dry diatom biomass extracted per mL cell or blood platelet suspension (mg DW mL^−1^).

### 4.3. Cell handling, experimental conditions and assessment of cell viability

Hepatocytes were isolated from male Wistar rats (80 to150 g) by *in vitro* collagenase perfusion [19,20]. They were resuspended (8.0 × 10^5^ cells mL^−1^) in pre-gassed (5% CO_2_/95% O_2_) 10 mmol L^−1^ Hepes buffer (pH 7.4) with 5 mmol L^−1^ lactate, 5 mmol L^−1^ pyruvate, 120 mmol L^−1^ NaCl, 5.3 mmol L^−1^ KCl, 0.01 mmol L^−1^ KH_2_PO_4_, 1.2 mmol L^−1^ MgSO_4_, and 1.0 mM CaCl_2_. For incubation with solvent (water or 25% aqueous DMSO) or extract, the cells were incubated (0.12 mL) in 48-well tissue culture dishes at 37 °C in 5% CO_2_ atmosphere with rotatory shaking (120 cycles min^−1^) for 60 min before adding fixative (2% formaldehyde in PBS, pH 7.4 with 0.5 mg mL^−1^ of the DNA-stain Hoechst 33342).

The IPC-81 rat leukemia cells [21] were cultured at 37 °C in a 5% CO_2_ atmosphere in DMEM medium supplemented with 10% horse serum. The experiments were performed as described above for the hepatocytes, except that the incubation was for 18 h.

The surface morphology was judged by differential interference microscopy and the degree of chromatin condensation by fluorescence microscopy, and the cells scored for apoptosis and necrosis as described previously [11,22]. The major criteria to judge if a cell was apoptotic were cell shrinkage, budding of the cell surface to produce protrusions that eventually detached to form apoptotic bodies, and chromatin hypercondensation. Apoptotic cells excluded Trypan blue. Necrotic cells underwent swelling and lost their ability to exclude Trypan blue.

For transmission electron microscopy the cells were fixed for 15 min in 1.5% glutaraldehyde in ice-cold Na-cacodylate buffer (pH 7.4), post-fixed for 60 min in 1% osmium tetraoxide and dehydrated by ethanol. After embedding the cells in Agar100 resin, ultra-thin sections were cut and stained in uranyl acetate and lead citrate. Sections were examined in a Philips EM 300/II electron microscope.

### 4.4. Preparation of human blood platelets and experimental conditions

Human venous donor blood (144 mL) was collected into 16 mL of anticoagulant (71 mmol L^−1^ citric acid, 85 mmol L^−1^ Na_3_-citrate, 122.1 mmol L^−1^ glucose). Concentrated platelet-rich plasma was prepared as described previously [23] and the platelets were further separated from plasma proteins by size-exclusion on a column packed with Sepharose CL-2B gel matrix (Pharmacia Biotec, Sweden). The platelets were eluted with Ca^2+^-free Tyrode’s buffer enriched with 0.1% BSA and 5.6 mmol L^−1^ glucose (pH 7.3), counted (Coulter Counter, Beckman Coulter UK Ltd, Bedfordshire, UK) and adjusted to 3.5 × 10^8^ platelets mL^−1^.

For measurement of platelet inhibition or activation, 2 μL of aqueous extract were added either 10 μL PBS or 10 μL adenosine deaminase (Sigma, CA, USA) in PBS (0.9 U mL^−1^) and incubated for 15 min before addition to 40 μL mixture of platelets and PE-conjugated anti-CD62 (BD biosciences, San Jose, CA, USA). After another 10-min incubation, 20 μmol L^−1^ thrombin receptor agonist peptide (TRAP, SFLLRN, Biotechnology centre of Oslo, Norway) was added and the platelets were left to activate for 15 min before fixation in 0.2% paraformalaldehyde in PBS. The ratio of PE-positive platelets (expressing P-selectin on the surface) was measured by a FACSCalibur (BD Biosciences, Franklin Lakes, NJ, USA) flow cytometer as previously described [24].

### 4.5. Extraction and isolation of bioactive material

Six grams of lyophilized dried diatom material from ND58 (*P. tricornutum*) was mixed with 350 mL of water at 4 °C, homogenized, 100 mL ice-cold water added and sonicated for 5 min in a sonication water bath, left for 2 h at 4 °C, and centrifuged at 14 000 × g for 10 min. The supernatant was subjected to anion exchange solid phase extraction (QMA 10 g, 35 cc Waters, Sep Pak®, Waters Corporation, Milford, MA, USA), conditioned with 100 mL of methanol and equilibrated with 200 mL of water (flow rate: 5–10 mL min^−1^). Each cartridge was loaded with sample corresponding to 1.5 g diatom dry mass, and washed with 75 mL water before elution with 100% methanol. The combined flow-through and water wash, and the methanol-fraction were collected separately and evaporated.

The load and wash fraction was further fractionated by semi-preparative RP-HPLC (Kromasil 100-5 C_18_ 250 × 10 mm column fitted with a guard column Kromasil 100-5 C_18_ 10 × 10 mm, both from AKZO Nobel, EKA Chemicals, Bohus, Sweden), with water (A) and acetonitrile (ACN, Rathburn, UK; B) as mobile phases, the gradient was: 0 min: 96% A and 4% B, 10 min: 84% A and 16% B, 24 min: 0% A and 100% B. The column was then washed with 100% B for 3 min. The flow rate was 4.5 mL min^−1^ and the monitoring wavelength was 238 nm. Fractions were collected every 30 s, evaporated in a vacuumed centrifuge, redissolved, and tested for cytotoxicity.

The cytotoxic fraction from the preparative column was evaporated and resuspended in 40 μL of water and injected onto an analytical C18 HPLC column (Kromasil 100-5 C_18_ 250 × 4.6 mm) fitted with a guard column (10 × 4.6 mm). Isocratic mobile phase mode (98 water, 2% ACN) enabled full resolution of the component peaks. The active peak was collected manually and further analysed by Q-tof MS and MSMS.

### 4.6. Q-TOF Ultima Global/Mass Spectrometry conditions

The spectrometer, Micromass Q-TOF Ultima Global, interface was operated in positive ion mode, capillary voltage: 3.00 kV, cone: 70 V, source temperature: 79, desolvation temperature: 100 °C, gas flow (L h^−1^) (a) cone: 50, (b) desolvation: 300. The purified diatom compounds and the adenosine and inosine standards were directly infused into the spectrometer at 1.0 μL min^−1^. Collision energies between 15 and 60 V were used to obtain fragments of the nucleosides.

### 4.7. Quantification of adenosine content in aqueous diatom extracts

Aqueous extracts were prepared as described for the crude extracts, and subjected to anion exchange solid phase extraction (QMA Sep-Pak® Light, Waters). The cartridges were conditioned with 2.0 mL methanol and equilibrated with 2.0 mL water. Volumes of extract corresponding to not more than 5 mg dry weight were loaded onto the columns followed by a wash with 2.0 mL MQ water and a final elution with 1.0 mL 20% methanol.

Ninety μL of the QMA-Load+Wash fractions were injected into the analytical HPLC (instrumentation as described above). The gradient was from 95:5% water-ACN to 85:15% water-ACN in 9 min, followed by 100% ACN to wash the column. The flow rate was 0.8 mL/min. Adenosine standards (20 μL) from 0.0012 mmol L^−1^ to 0.08 mmol L^−1^ were chromatographed under identical conditions to create a standard curve. The adenosine peak was identified by retention time and UV-spectrum, and the area under the peak was used to determine the adenosine concentration.

For the stereo-specific enzymatic degradation of adenosine four units of adenosine deaminase were added to adenosine (1 mM) or the bioactive 11.9-min peak (Figure 3) to a final volume of 0.1 mL, and the mixtures allowed to react for 30 s before stopping the reaction by adding 50 μL of ACN. The product was added to either adenosine or Apo-58-11, and the mixtures were chromatographed.

### 4.8. DNA accession numbers of diatom isolates

Partial sequences from genomic DNA of LSU (28S) from the diatom isolates have been deposited in GenBank (at http://www.ncbi.nlm.nih.gov/) with the following accession numbers: FJ214161(ND51); FJ214162(ND52); FJ214163(ND53); FJ214164(ND55); FJ214165(ND56); FJ214166(ND58); FJ214167(ND 71); FJ214168(ND73); FJ214169(ND79).

## 5. Supplementary data

### 5.1. Mass cultivation of the diatom isolate ND58

Large batch cultures were grown in Conway medium [18] prepared from filtrated seawater diluted with distilled water to 80% at 20 °C. Humified air mixed with pure CO_2_ to obtain 1% CO_2_ was filtered (0.45 μm) and bubbled through the cultures, and a magnetic stirrer at the bottom prevented sedimentation of the cells. The 10 L culture vessels were polycarbonate cylinders (height 110 cm, inner diameter 11 cm). Cultures were provided with 250 μmol photons m^−2^ sec^−1^ continuous light from fluorescent tubes (Phillips). Scalar irradiance was measured at the front of the cultures with a spherical sensor (Biosperical Instruments Inc. QSL-100). Cultures were harvested in early stationary growth phase after one week by continuous centrifugation at 1,000 rpm, washed with MQ water (MilliQ quality, Millipore system, Millipore Billerica, MA), frozen and lyophilized. Approximately 6 g wet weight was typically obtained from 10 L cultures.

### 5.2. Taxonomy and phylogeny of diatom isolates

#### 5.2.1. Light microscopy

Live cultures were studied in a Leica DMRBE microscope. Measurements of cell dimensions (n = 20) and observations of cell shape and chain formation of cells were mainly based on light microscopy at 400× magnification.

#### 5.2.2. Scanning electron microscopy

The most characteristic feature of the diatoms is the siliceous cell wall that is made up of two almost equal halves, and together they form the frustule. This siliceous frustule can have highly detailed patterns or ornamentation, and these features are important in diatom taxonomy.

Diatom frustules were cleaned with potassium permanganate and sulfuric acid as described [25]. Samples were then carefully filtrated onto polycarbonate membrane filters (0.6 μm pore size, Poretics^®^), rinsed with distilled water on the filter and dried in a desiccator for two days. The oval *Phaeodactylum tricornutum* cells (isolate ND 52, 55, 56, 58) have only one silicified cell wall is degraded under standard preparation methods. These cells were conserved in 2% glutaraldehyde, before filtered as described above. All samples were coated with gold-palladium and studied using a scanning electron microscope (JEOL SEM 6400, Tokyo, Japan).

The frustules were sized for width (transapixal axis) and length (apixal axis) and analyzed for density of striae, patterns of perforations, presence of raphe and for other details of the frustules. The terminology used to describe the diatom frustules follows [26] and [27].

The ND50 isolate was studied by light microscopy of live cells. The cells were close together in chains of 10 to 40 cells united by mucilage pads. The valve diameter was 7.5 μm and the valve height varied from 7.5 to 12.5 μm. The valve mantles were strongly curved, and these characteristics correlates with features described for the genus *Melosira* C. A. Agardh 1824 [28].

**Figure S1.**
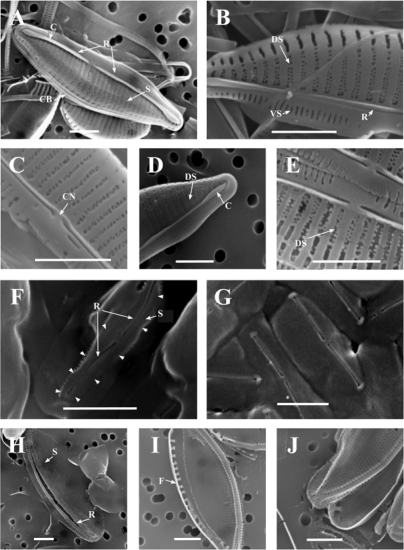
Scanning electron micrographs of frustules of diatom isolates. A to C: *Amphora cf. margalefii* (ND 51). B: Note the double row of puncta in striae on dorsal side. C: Detail of central area. Note small central nodule. D and E: *Amphora* sp. (ND 79). F and G: *Phaeodactylum tricornutum* (ND 58). In F, note the partially silicified valve. The arrows indicate its margins. H to J: *Nitzschia cf. pusilla* (ND 73). H: Note the unbroken raphe from pole to pole. In J we see grater detail of striation.C-conopeum; CB-connecting bands; CN-central nodule; DS-Dorsal striae; F-Fibulae; R-Raphe; S-Striae; VS-Ventral striae. Scale bars represent 2 μm.

Representative presentations of SEM micrographs of frustules from the different isolates are presented in Figure S1 (A to J). The morphology of ND51 (Figure S1 A–C) corresponds well with the description of *Amphora margalefii* Tomás sp. nov. [29], and the ND51 isolate was thus tentatively identified as *Amphora cf. margalefii*.

ND71 and ND79 had identical morphological features (see Figure S1 D and E for images of ND79). These two strains differed from ND51 only in the dorsal striae density and cell size (ND71 and 79: dorsal striae density: 35 in 10 μm, size: length: 17.5–20 μm width 5–10 μm. ND51: dorsal striae density 25 in 10 μm, size: length 10–15 μm, width 5–6.25 μm). Based on these differences we conclude that ND51 should be separated from ND71 and ND79. The difference at a generic level confirms this. The identity of ND71 and ND79 is not certain, but it is close to ND51, and it can be a similar species like *A. delicatissima* Krasske [30] or maybe a new species.

Morphological features from LM and SEM observations of ND 52, 55, 56 and 58 isolates were identical and corresponded well with the description of the oval cell form of *Phaeodactylum tricornutum* Bohlin 1897 emend Lewin 1958 [30] (Figure S1 F and G). Lewin (1958) established a new suborder *Phaeodactylineae* on basis of the unique characters mainly due to the atypical partially silicified cell wall and its polymorph nature. The siliceous valve is naviculoid and occurs on only one side of the cell wall [30,31]. In Figure S1 F one can see the siliceous part on one valve protruding from the organic layer, and the striae density was approximately 95 per 10 μm. In Figure S1 F and G the raphe slits are visible trough the organic layer surrounding the frustules, and the oval cell form can be seen. The lengths of these cells were from 5 to 8 μm and the widths were 2.5 to 3.5 μm. When these isolates were cultured in liquid cultures small fusiform cells were occasionally observed in LM, a morphological plasticity that is characteristic for *P. tricornutum*. The genomic and morphological identification of these isolates correlated, and ND52, 55, 56 and 58 were identified as *P. tricornutum*.

The morphological features of silica frustules of ND53 and ND73 corresponded with features described for *Nitzschia pusilla* Grunow 1862 emend. Lange-Bertalot 1976 [32]. Cells are elliptic to linear-lanzeolate. The lengths of the cells were 10 to 15 μm, and the widths were 3.7 to 6.3 μm. The raphe was unbroken from pole to pole (Figure S1 H). Striae density was 54 per 10 μm. The densities of fibulae were from 20 to 25 per 10 μm (I and J). These findings and the 100 % match with partial LSU sequences identified ND53 and ND73 as *Nitzschia cf. pusilla* (emend Lange-Bertalot).

#### 5.2.3. DNA extraction and amplification of parts of LSU rDNA

Cells from 10 mL from each algae culture were sampled by gentle centrifugation and frozen. Genomic DNA was extracted using DNeasy® Plant Mini Kit (QIAGEN). Initially sterilized fine-grained sand was used together with a pestle in the Eppendorf tube to open the cells properly, and the mixture was resuspended in lysis buffer as described in the kit. The PCR mix was described in [33] and a 50 μL PCR reaction was used.

The PCR primers (D1R-F [34] and D3B-R [35]) were purchased from Eurogentec (EGT group, USA). PCR amplification conditions were: one initial denaturation at 94 °C for 2 min followed by 35 cycles each consisting of 1 min at 92 °C, 55 °C for 1 min, 72 °C for 1 min and finally 72 °C for 7 min.

Sequencing of PCR products: The PCR products were purified using ExoSAP-IT® (USB, USA). Ten to twenty ng of the PCR product were used in each 10 μL sequence reaction, and the PCR products were sequenced in both directions. Sequencing primers were the two PCR amplification primers and BigDye terminator cycle sequencing kit was used (Version 3.1, Applied Biosystems).

#### 5.2.4. Alignment and analyses of sequences

**Figure S2.**
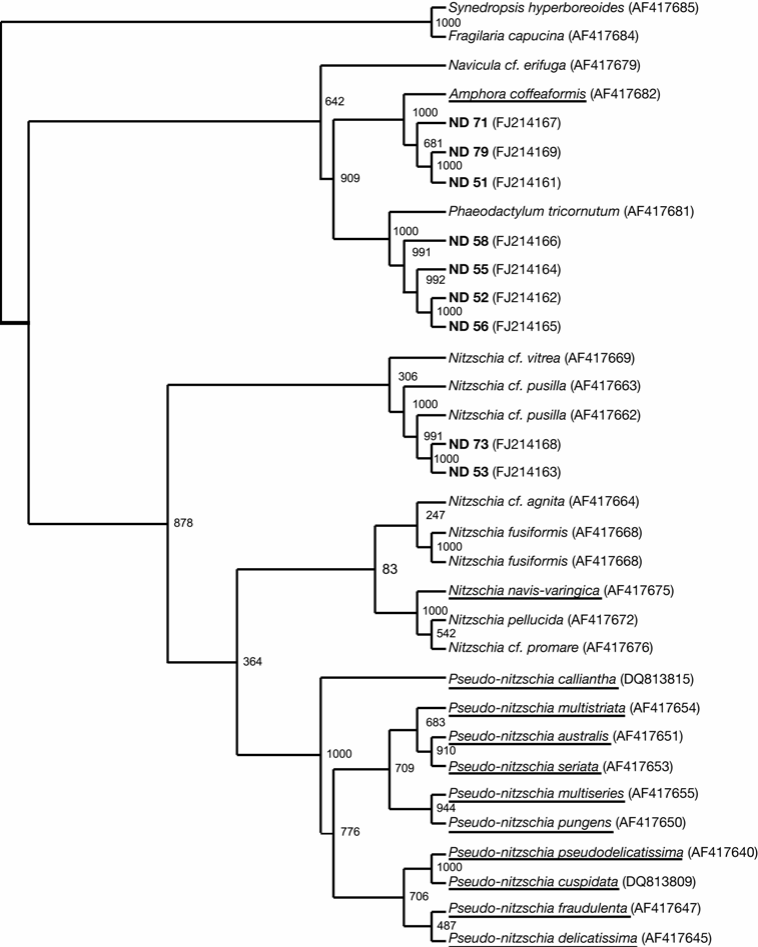
Neighbour-joining tree from partial LSU sequences of domoic acid producing diatoms (underlined), other pennate diatoms and our isolates. The three was bootstrapped 1000 times and values are shown at the nodes. The araphide species *Synedropsis hyperboreoides* and *Fragilaria capucina* were used to root the three.

Sequences were edited and aligned manually in BioEdit [36], and Blastn 2.2.18 [37] was used to search for similarities with previously published diatom LSU rDNA in GenBank [33]. The production of domoic acid is the best-described bioactivity in diatoms. Therefore sequences from partial LSU from our isolates were aligned with diatom species that are previously known to produce domoic acid. Sequences from partial LSU of domoic producing diatoms (underlined in Figure S1) were multiple aligned with our isolates using Clustal W [38]. A neighbour-joining tree based on 1000 bootstrap values was calculated in Clustal X [39] and finalized in tree view [40], and bootstrap values were included at the nodes of the tree (Figure S2). *Synedropsis hyperboreoides* and *Fragilaria capucina* (araphide species) were included as outgroups to root the tree. Accession numbers in GenBank for our isolates and the included sequences are presented in brackets in Figure S2.

We examined how our isolates grouped with previously known domoic acid producers in a phylogenetic tree constructed by the neighbour-joining method (Figure S2). Our isolates did not cluster with the most common domoic acid producers like *Pseudo-nitzschia* sp. and *Nitzschia* sp. However, isolate ND 51, ND 71 and ND 79, belonging to the *Amphora* genus, clustered with the lesser common domoic acid producer *Amphora coffeaformis*. It should be noted that domoic acid is a neurotoxin, and would not be detected in our bioassays. We did not make any attempt to identify domoic acid in our isolates.

## Figures and Tables

**Table 1.** Strain code, sampling site and toxicity of the diatom isolates.

**Isolate no.**	**Geographical origin of isolates**	**Toxicity**								
		**Hepatocytes**			**IPC-81wt**			**IPC-Bcl-2**		

		**A**	**B**	**C**	**A**	**B**	**C**	**A**	**B**	**C**
ND50	Odda	−	−	−	**++**	**+**	−	−	−	−
ND51	Masfjord	−	**+**	−	**+**	**+**	−	−	−	−
ND52	Puddefjorden	−	−	−	**++**	**+**	−	**++**	−	−
ND53	Utne	−	−	**+**	**+**	**+**	−	−	−	−
ND55	Masfjord	−	−	−	**++**	**+**	−	**+**	−	−
ND56	Odda	−	−	−	**++**	−	−	**+**	−	−
ND58	Puddefjorden	+	−	−	++	−	−	+	−	−
ND71	Puddefjorden	**+**	**+**	−	**+**	**+**	−	−	−	−
ND73	Tromøy	**+**	−	−	++	**+**	−	**+**	−	−
ND79	Puddefjorden	−	**+**	−	**++**	**+**	−	−	−	−

A: aqueous extract, B: 70% aqueous methanol extract, C: 1:1 dichloromethane:methanol extract. The symbols on the toxicity data signify: –: no toxicity; +: more than 30% cell death at 4 mg DW mL^−1^; ++: more than 30% cell death at 1.5 mg DW mL^−1^. Incubation times were 1 h for hepatocytes and 18 h for IPC-cells.

**Table 2.** Taxonomy, adenosine content and platelet modulatory activity of the diatom isolates.

**Isolate no.**	**Taxonomy of isolates**	**Adenosine content, μg ado/mg DW**	**Modulation of TRAP-induced platelet activation**	
			**Adenosine deaminase**	
			**Without**	**With**
ND50	*Melosira* sp.	0.21	---	---
ND51	*Amphora cf. margalefii* Tomás sp. nov.	0.07	---	--
ND52	*Phaeodactylum tricornutum*	0.16	--	---
ND53	*Nitzschia cf. pusilla*	<0.05	+	+
ND55	*Phaeodactylum tricornutum*	0.18	---	---
ND56	*Phaeodactylum tricornutum*	0.19	---	---
ND58	*Phaeodactylum tricornutum*	0.17	---	---
ND71	*Amphora cf. delicatissima* or new *Amphora* sp.	<0.05	--	-
ND73	*Nitzschia cf. pusilla*	0.31	---	○
ND79	*Amphora cf. delicatissima* or new *Amphora* sp.	0.15	---	○
*P. tricornutum*, CCAP		0.11		

Blood platelet inhibition: ---, --, -: < 25%, <50%, and <75% P-selectin expression, respectively, compared to control. Blood platelet activation: +: >200% P-selectin expression compared to control. ○: Less than 25% change in P-selectin expression. The blood platelets were incubated with aqueous extracts at 4 mg DW mL^−1^.
